# The Potential Importance of MicroRNAs as Novel Indicators How to Manage Patients with Juvenile Idiopathic Arthritis More Effectively

**DOI:** 10.1155/2021/9473508

**Published:** 2021-01-28

**Authors:** Krzysztof Orczyk, Elzbieta Smolewska

**Affiliations:** Department of Pediatric Cardiology and Rheumatology, Medical University of Lodz, Sporna 36/50, 91-738 Lodz, Poland

## Abstract

Small, noncoding sequences of ribonucleic acid called microRNAs (miRNAs, miR) are functioning as posttranscriptional regulators of gene expression. As they draw increasing attention of rheumatologists, there is a growing body of evidence concerning specific molecules that may affect the long-term care of patients with inflammatory arthritides. Findings involving children with juvenile idiopathic arthritis (JIA) are still limited though. The aim of the study was to browse the available data on microRNAs which may be utilized as potential biomarkers helpful in diagnosing and monitoring JIA patients. The review contains a brief summary on the most studied sequences: miR-16, miR-125a-5p, miR-146a, miR-155, and miR-223. It is complemented with other miRNAs possibly relevant for JIA (miR-145, miR-23b, miR-27a, and miR-204) and discussion on challenges for using miRNAs in pediatric rheumatology (particularly, issues regarding specificity of biomarkers and measurements involving synovial fluid).

## 1. Introduction

**Figure 1:**
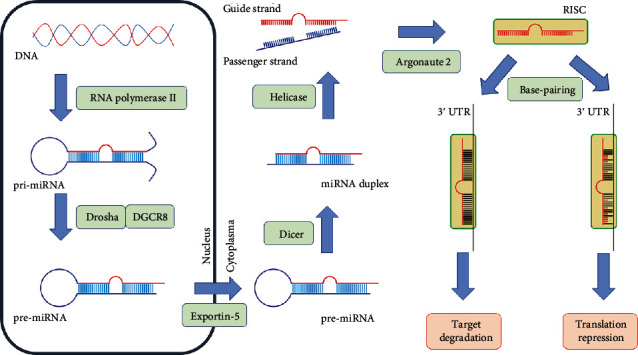
Biogenesis and functioning of miRNA. The figure illustrates the following steps: (a) endonuclear synthesis of the primary double-stranded miRNA transcript (pri-miRNA) of hundreds to thousands of nucleotides in length, mostly by RNA polymerase II; (b) cleavage of pri-miRNA by Drosha endonuclease accompanied by DiGeorge syndrome critical region protein 8 (DGCR8), resulting in formation of 60 to 70 nucleotide long pre-miRNA; (c) export of pre-miRNA from the nucleus into the cytoplasm by Exportin-5 coacting with GTP-binding nuclear protein Ran (Ran-GTP); (d) cleavage of pre-miRNA by Dicer into an asymmetric miRNA duplex containing guide and passenger strand of about 22 nucleotides in length; (e) unwinding the duplex by helicase and release of the passenger miRNA with its subsequent degradation; (f) loading the guide strand by Argonaute 2 into the RNA-induced silencing complex (RISC); (g) binding RISC (containing mature miRNA) to 3′ untranslated region (3′ UTR) of target mRNA; (h) translational repression or target degradation depending on the partial or full complementarity of base-pairing. Based on [60–62].

Small, noncoding sequences of microRNA (miRNA, miR) have already established their position as an innovative part of the diagnostic process in the bulk of disorders, with particular emphasis placed on neoplastic diseases. miRNAs should be considered as negative posttranscriptional regulators affecting the expression of genes involved in both innate and adaptive immune responses [1]. Karami et al. stated that nearly 60% of human genes contain at least one binding site for miRNA [2]. By direct base-pairing with the 3′ untranslated region (3′ UTR) of target mRNAs [3], miRNAs can suppress or impair gene translation leading to the inhibition of target protein synthesis [4]. A brief summary of miRNA biogenesis and its functioning is depicted in Figure 1.

According to Ormseth et al. [5], miRNAs are not limited to intracellular activity, but they can also be transported to recipient cells through plasma, where they are protected from degradation by several mechanisms, involving exosomes, microvesicles, lipoproteins, and RNA-binding proteins. High robustness of cell-free miRNAs in body fluids (e.g., plasma and serum) supports their potential role as reliable biomarkers [6, 7]. However, concentrations of particular miRNAs may differ from each other depending on the source of the examined material. Interestingly, miR-132, which has been observed to be significantly decreased in serum of RA patients, turned out to be overexpressed in synovial PBMCs [8].

Growing popularity and availability of measuring these molecules reflects from PubMed statistics: only in 2019, the number of the registered papers regarding miRNA exceeded 15,000 articles. Of these, just 121 referred to adult patients with rheumatoid arthritis (RA), whereas juvenile idiopathic arthritis (JIA) was involved in merely eight studies. The first study which postulated the role of miRNA in the pathogenesis of RA dates back to 2007, when Bhanji et al. reported that sera of RA patients include antibodies against Argonaute 2 (a protein required for miRNA-mediated gene silencing) [9]. In the following years, Romo-Garcia et al. speculated that the transcriptional arrest present at the early stages of RA may be related to overexpression of miRNAs and subsequent intensified inhibition of translation [7]. The initial evidence regarding miRNA in JIA, specifically miR-155, was reported in 2014 [10]. A thorough literature review involving the PubMed database provided at least 55 miRNA sequences (listed and briefly described in Supplementary Table 1) which may be applicable to rheumatic diseases. They may affect the pathogenesis of JIA through several mechanisms which include, among others, repressing the expression of genes that are crucial for the TNF/IL-1 pathway [11] or dysregulating the macrophage polarization [12]. Within this paper, we will summarize the available data on the most studied miRNAs in RA and JIA (miR-16, miR-125a-5p, miR-146a, miR-155, and miR-223). Then, we will shed a light on several less known sequences which bear a potential for the future diagnostic process and monitoring disease activity in JIA patients.

## 2. miR-16

miR-16 seems to be one of the crucial factors for the pathogenesis of RA and JIA, as through targeting the silencing mediator for retinoid and thyroid hormone receptor (SMRT), it regulates the expression of proinflammatory cytokines, particularly interleukin- (IL-) 1*α*, IL-6, and IL-8 [13]. Zhou et al. [14] hypothesized the role of miR-16 as a key regulator of Toll-like receptor-signaled inflammatory response. According to Murata et al. [15], miR-16 levels detected in RA patients were markedly higher than in osteoarthritis patients. Furthermore, Demir et al. [16] reported elevated miR-16 concentrations in JIA patients when compared to healthy controls. Nevertheless, the plasma level of this molecule fluctuates in a disease activity-dependent manner which may lead to contradictory findings varying on the measurement protocol. Hence, miR-16 was significantly upregulated in children at the onset of both poly- and oligoarticular JIA [17], but it inversely correlated with Disease Activity Score 28-Joint Count (DAS28) in patients with the average RA duration of 10.4 years [15]. Interestingly, Filková et al. [18] observed that miR-16 is higher in patients with established RA than in the early stage of the disease. Moreover, they hypothesized that this molecule may be utilized as a biomarker of RA outcome because its pretherapy level correlated with disease activity drop after 9 months of treatment. Correlation between high disease activity and miR-16 concentrations was also observed in inflammatory bowel diseases [19] and in ankylosing spondylitis [20]. Nonetheless, children with juvenile spondyloarthropathy had lower miR-16 concentrations than JIA patients [17]. Regarding JIA, miR-16 tends to have higher values in poly- rather than in oligoarthritis [17]. However, the discrepancy observed between subtypes was insufficient to differentiate patients basing only on that parameter.

## 3. miR-125a-5p

Research in rheumatology on miR-125a-5p involved mainly patients with systemic onset of JIA (SoJIA), which is characterized by autoinflammatory rather than autoimmune response. Monocytes isolated from children in the active phase of SoJIA abundantly overexpressed miR-125a-5p when compared to patients in clinical remission or with active polyarthritis [21]. Moreover, Do et al. [22] noted that miR-125a-5p markedly restricted activation of macrophages' anti-inflammatory phenotype involving expression of CD163. Furthermore, Leong et al. [23] observed a positive correlation between miR-125a-5p and indicators of systemic inflammation (particularly ferritin) with no parallel relation to joint involvement. However, due to the distinct pathogenesis of SoJIA, findings from this subtype cannot be directly extrapolated to all JIA patients. Evangelatos et al. [24] reported increased miR-125a-5p levels in early RA patients, regardless of the results of “classic” serological markers (rheumatoid factor and anticitrullinated protein antibodies). It is also worth interjecting here that miR-125b, which shares the seed sequence and target specificity with miR-125a-5p [25], was the first microRNA recognized as a predictor of treatment outcome in RA patients (specifically, response to rituximab) [26]. Therefore, the final position of measuring miR-125a-5p in JIA patients with active joint inflammation merits further investigation.

## 4. miR-146a

The pathogenetic role of miR-146a in developing inflammatory arthritis is expressed through several pathways of insufficient negative feedback [27]. By targeting inhibin beta A (INHBA), it regulates differentiation of macrophages [28] towards M2 type [29]. It also targets IL-1 receptor-associated kinase 1 and 2 (IRAK1, IRAK2) as well as tumor necrosis factor (TNF) receptor-associated factor 6 (TRAF6) [30], which enables suppression of NFkB signaling [3] and modulation of TNF production [31]. Furthermore, miR-146a promotes the expression of proinflammatory cytokines (including, among others, IL-2 and IL-12) by its upregulation in Th1 [32] and Th17 lymphocytes [24] with concurrent downregulation in Th2 cells [33]. Despite being decreased in early RA when compared to the patients with established diagnosis [34], miR-146a was found to be elevated in oligo-, polyarticular, and SoJIA [17, 28]. Besides, the single-nucleotide polymorphism (SNP) of miR-146a sequence was postulated to be associated with susceptibility to develop enthesitis-related arthritis (JIA-ERA) [35] and ankylosing spondylitis [36]. Concerning disease activity, miR-146a positively correlated with DAS28 [15] and Juvenile Arthritis Disease Activity Score 27-Joint Count (JADAS27) [17]. Moreover, Li et al. [28] found miR-146a levels to be associated with the systemic features. Additionally, the molecule has also been assessed as a good predictor of response to methotrexate therapy (AUC 0.760 in the study conducted by Singh et al. [37]).

## 5. miR-155

Through targeting the suppressor of cytokine signaling 1 (SOCS1), miR-155 acts as a key promoter of M1 macrophages [38]. At the same time, it directly affects IL-13 receptor alpha 1 (IL-13R*α*1) which results in the inhibition of M2 activation [39]. Besides, Kurowska-Stolarska et al. found the significant expression of miR-155 in CD68+ macrophages within the lining layer of the synovial membrane in RA patients [40]. Furthermore, miR-155 enhances the production of IL-2 by peripheral blood mononuclear cells (PBMCs) [10]. Additionally, Vigorito et al. [41] reported the impact of miR-155 on B cell maturation into class-switched plasma cells releasing immunoglobulin G1. Interestingly, transcriptional alterations detected in neutrophils isolated from plasma of children with JIA seem to perpetuate regardless of the disease activity [42]. Demir et al. [16] found that upregulation of miR-155 was even more significant in JIA patients with the improvement in disease activity after 6 months of therapy. However, in adults with ankylosing spondylitis, the elevated miR-155 levels correlated with an increase in disease activity index [43]. miR-155 concentrations tended to be higher in children with polyarthritis, but the difference did not reach statistical significance [16]. Moreover, Ma et al. [17] obtained similar miR-155 values in patients diagnosed with JIA or juvenile spondyloarthropathy which further diminished its specificity. As summarized by Su et al. [44], overexpression of miR-155 in RA patients correlated with the inflammatory markers, DAS28 as well as with TNF and IL-1*β* levels. Similar to miR-146a, miR-155 appeared to be a promising prognostic marker of methotrexate effectiveness (AUC 0.728 in the same study [37]).

## 6. miR-223

The upregulation of miR-223 in peripheral naïve CD4+ T lymphocytes [45] plays a pivotal role in the development of local joint inflammation. Given that miR-223 is involved in the activation of M2 macrophages through targeting PBX/Knotted 1 Homeobox 1 (Pknox 1) [46] and in the suppression of nucleotide-binding oligomerization domain-like receptor protein 3 (NLRP3) inflammasome activity [47], its detected overexpression may be hypothesized as an indicator of malfunctioning inflammatory response. Intriguingly, miR-223 applies not only to the articular manifestations, as the elevated serum levels of the molecule were found also in children with SoJIA [6]. Moreover, miR-223 concentrations were associated with CRP levels [18] and titer of rheumatoid factor (RF) [48] in RA patients. It also appeared to be a reliable predictor of change in disease activity within a 3- and 12-month observation [18]. Furthermore, elevated miR-223 values before treatment initiation were directly correlated with better response to methotrexate and inversely correlated with anti-TNF efficacy [24]. miR-223 seems to be the first epigenetic factor that was speculated as a novel RA therapeutic target by Li et al. [49], who observed that miR-223 inhibition reduced the disease severity of murine collagen-induced arthritis.

## 7. Other miRNAs Possibly Relevant for JIA

None of the aforementioned, widely studied miRNA sequences were characterized as being specific to oligoarticular JIA, which is considered as the most common subtype of the disease. Nevertheless, Sun et al. [50] noted that miR-145 is upregulated in oligoarthritis comparing to polyarthritis and SoJIA. Besides, miR-23b appeared to be overexpressed in RA patients with the relevant titer of antinuclear antibodies (ANA) [51]. Given that the variability of gene expression may be correlated with a child's age at disease onset [52], such findings may contribute to the task of defining early onset ANA-positive arthritis which is planned to be discerned in the upcoming reclassification of JIA [53].

Current approach to JIA therapy includes prompt administration of biological agents in patients who did not respond to the first-line treatment, namely, methotrexate [54]. Interestingly, miR-27a may serve as an efficient predictor of whether a patient is likely to improve on anti-TNF therapy. Increased initial miR-27a levels in adults with early RA were associated with better response to a combination therapy of methotrexate with adalimumab, particularly when the concentrations of the molecule decreased within the first 3 months of such treatment [55]. On the other hand, suspending methotrexate in JIA children that achieved clinical remission on medication is another frequent concern of pediatric rheumatologists. Nonetheless, miR-204 turned out to be markedly decreased in patients with inactive disease who continue methotrexate in monotherapy [16]; therefore, it may become a biomarker helpful in therapeutic decisions.

## 8. Challenges for miRNA in Pediatric Rheumatology

JIA is the most common arthropathy in childhood, though it is not characterized by homogenous natural history of the disease. Patients differ from each other with regard to the disease subtype, number and location of the inflamed joints, time of developing new symptoms and complications, and treatment response. One of the major objectives of research in pediatric rheumatology is to seek for and determine effective markers (mainly serological and genetic) which may facilitate prognosing the disease course and therefore optimize JIA treatment. However, insufficient specificity is a frequent drawback of biomarkers utilized to measure the activity of inflammation. The most studied serological biomarkers in JIA include S100 proteins, particularly S100A8/A9 [56] and S100A12 [57], which are very sensitive in detecting high disease activity. Nevertheless, chronic neutrophil activation may be observed as well in other entities with a distinct origin. For instance, Hu et al. obtained similar S100A8/A9 values in both JIA and cystic fibrosis patients [42]. miRNAs as a class of molecules are not free of such limitations. Despite the potential usefulness of miR-23b in diagnosing rheumatic diseases, this molecule is also related to fibrogenesis within chronic liver injury and diabetic nephropathy [51]. Nevertheless, there are several miRNAs which have already been tested whether they are specific for systemic autoimmune diseases. Jin et al. reported three sequences (namely, miR-124, miR-448, and miR-551b) which had altered values in patients with RA, systemic lupus erythematosus, Sjögren's syndrome, and ulcerative colitis; the authors did not find significant differences of the miRNAs' levels between healthy controls and patients with pneumonia, sepsis, and HBV hepatitis [58].

There are several studies underlining the importance of a distinct route of miRNA expression in fibroblast-like synoviocytes (FLS) [15, 59]. Nziza et al. reported a set of miRNAs extracted from the synovial fluid (miR-6764-5p, miR-155, and miR-146a) that perfectly distinguish (AUC 1.0) children with JIA from patients with *Kingella kingae* septic arthritis [60]. However, such proceedings are more invasive than the examination of blood samples and exclude the possibility of comparison with healthy controls due to ethical issues. Therefore, further investigation of miRNAs in JIA should focus on the sequences that may ease the differentiation of patients basing on plasma and/or serum levels.

## 9. Conclusions

miRNAs seem to open a new chapter of diagnosing and monitoring activity of childhood arthropathies on the epigenetic level. They might provide the probable answer which patients are more likely to develop a more aggressive course of disease or respond properly to the administered therapy. The authors recommend to continue validation of the already well-known sequences (miR-16, miR-125a-5p, miR-146a, miR-155, and miR-223) and to further investigate the alternative promising molecules (miR-145, miR-23b, miR-27a, and miR-204). Studies including patients that are supposed to be recognized as early onset ANA-positive JIA are of utmost importance.

## Data Availability

The data used to support the findings of this study are included within the article.

## Supplementary Materials

Supplementary Materials Supplementary Table 1: list of microRNAs studied in rheumatology. ACPA: anti-citrullinated protein antibodies; ADA: adalimumab; ANA: antinuclear antibodies; AOSD: adult-onset Still's disease; AS: ankylosing spondylitis; BTRC: beta-transducin repeat containing gene; CD: cluster of differentiation; CF: cystic fibrosis; CIA: collagen-induced arthritis; CRP: C-reactive protein; DAS28: Disease Activity Score 28-Joint Count; DMARD: disease-modifying antirheumatic drug; ERA: enthesitis-related arthritis; ESR: erythrocytes sedimentation rate; FLS: fibroblast-like synoviocytes; foxp3: forkhead box P3; FMF: familial Mediterranean fever; GCS: glucocorticosteroids; IL: interleukin; IFN*β*: interferon beta; INHBA: inhibin beta A; IRAK: interleukin-1 receptor-associated kinase; JADAS27: Juvenile Arthritis Disease Activity Score 27-Joint Count; JAK/STAT: Janus kinase/signal transducers and activators of transcription; JDM: juvenile dermatomyositis; JIA: juvenile idiopathic arthritis; JSA: juvenile spondyloarthropathy; miR: microRNA; MMP: matrix metalloproteinase; MRI: magnetic resonance imaging; MTX: methotrexate; NLRP3: NLR family pyrin domain containing protein 3; NFkB: nuclear factor kappa of activated B cells; OA: osteoarthritis; PBMC: peripheral blood mononuclear cell; PLT: platelet count; PsA: psoriatic arthritis; PTGS2: prostaglandin-endoperoxide synthase 2; RA: rheumatoid arthritis; RF: rheumatoid factor; RORyt: retinoic-acid-receptor-related orphan nuclear receptor gamma; RTX: rituximab; SLE: systemic lupus erythematosus; SNP: single-nucleotide polymorphism; SOCS3: suppressor of cytokine signaling 3; SoJIA: systemic onset of juvenile idiopathic arthritis; SS: Sjögren syndrome; SSZ: sulfasalazine; TAK1: transforming growth factor-beta-activated kinase 1; Th: helper T cells; TLR: Toll-like receptor; TNF: tumor necrosis factor; TRAF6: tumor necrosis factor receptor-associated factor 6; Treg: regulatory T cells; UC: ulcerative colitis; WBC: white blood count.
