# Supplementary material for: The Potential Importance of MicroRNAs as Novel Indicators How to Manage Patients with Juvenile Idiopathic Arthritis More Effectively
Source: J Immunol Res. 2021 Jan 28;2021:9473508. doi: 10.1155/2021/9473508 (PMC7864733; doi:10.1155/2021/9473508)
Supplement: Supplementary Materials — Supplementary Table 1: list of microRNAs studied in rheumatology. ACPA: anti-citrullinated protein antibodies; ADA: adalimumab; ANA: antinuclear antibodies; AOSD: adult-onset Still's disease; AS: ankylosing spondylitis; BTRC: beta-transducin repeat containing gene; CD: cluster of differentiation; CF: cystic fibrosis; CIA: collagen-induced arthritis; CRP: C-reactive protein; DAS28: Disease Activity Score 28-Joint Count; DMARD: disease-modifying antirheumatic drug; ERA: enthesitis-related arthritis; ESR: erythrocytes sedimentation rate; FLS: fibroblast-like synoviocytes; foxp3: forkhead box P3; FMF: familial Mediterranean fever; GCS: glucocorticosteroids; IL: interleukin; IFNβ: interferon beta; INHBA: inhibin beta A; IRAK: interleukin-1 receptor-associated kinase; JADAS27: Juvenile Arthritis Disease Activity Score 27-Joint Count; JAK/STAT: Janus kinase/signal transducers and activators of transcription; JDM: juvenile dermatomyositis; JIA: juvenile idiopathic arthritis; JSA: juvenile spondyloarthropathy; miR: microRNA; MMP: matrix metalloproteinase; MRI: magnetic resonance imaging; MTX: methotrexate; NLRP3: NLR family pyrin domain containing protein 3; NFkB: nuclear factor kappa of activated B cells; OA: osteoarthritis; PBMC: peripheral blood mononuclear cell; PLT: platelet count; PsA: psoriatic arthritis; PTGS2: prostaglandin-endoperoxide synthase 2; RA: rheumatoid arthritis; RF: rheumatoid factor; RORyt: retinoic-acid-receptor-related orphan nuclear receptor gamma; RTX: rituximab; SLE: systemic lupus erythematosus; SNP: single-nucleotide polymorphism; SOCS3: suppressor of cytokine signaling 3; SoJIA: systemic onset of juvenile idiopathic arthritis; SS: Sjögren syndrome; SSZ: sulfasalazine; TAK1: transforming growth factor-beta-activated kinase 1; Th: helper T cells; TLR: Toll-like receptor; TNF: tumor necrosis factor; TRAF6: tumor necrosis factor receptor-associated factor 6; Treg: regulatory T cells; UC: ulcerative colitis; WBC: white blood count. [file 9473508.f1.pdf]

Supplementary Table 1. List of microRNAs studied in rheumatology.

ACPA – anti-citrullinated protein antibodies, ADA – adalimumab, ANA – antinuclear antibodies, AOSD – Adult-onset Still's disease, AS – ankylosing spondylitis, BTRC – beta-transducin repeat containing gene, CD – cluster of differentiation, CF – cystic fibrosis, CIA – collagen-induced arthritis, CRP – C-reactive protein DAS28 – Disease Activity Score 28-Joint Count, DMARD – disease-modifying anti-rheumatic drug, ERA – enthesitis-related arthritis, ESR – erythrocytes sedimentation rate, FLS – fibroblast-like synoviocytes, foxp3 – forkhead box P3, FMF – Familial Mediterranean Fever, GCS – glucocorticosteroids, IL – interleukin, IFNβ – interferon beta, INHBA – inhibin beta A, IRAK – interleukin-1 receptor-associated kinase, JADAS27 – Juvenile Arthritis Disease Activity Score 27-Joint Count, JAK/STAT – Janus kinase/signal transducers and activators of transcription, JDM – juvenile dermatomyositis, JIA – juvenile idiopathic arthritis, JSA – juvenile spondyloarthropathy, miR – microRNA, MMP – matrix metalloproteinase, MRI – magnetic resonance imaging, MTX – methotrexate, NLRP3 – NLR family pyrin domain containing protein 3, NFkB – nuclear factor kappa of activated B cells, OA – osteoarthritis, PBMC – peripheral blood mononuclear cell, PLT – platelet count, PsA – psoriatic arthritis, PTGS2 – prostaglandin-endoperoxide synthase 2, RA – rheumatoid arthritis, RF – rheumatoid factor, RORyt – retinoic-acid-receptor-related orphan nuclear receptor gamma, RTX – rituximab, SLE – systemic lupus erythematosus, SNP – single-nucleotide polymorphism, SOCS3 – suppressor of cytokine signaling 3, SoJIA – systemic onset of juvenile idiopathic arthritis, SS – Sjögren syndrome, SSZ – sulfasalazine, TAK1 – transforming growth factor-beta-activated kinase 1, Th – helper T cells, TLR – Toll-like receptor, TNF – tumor necrosis factor, TRAF6 – tumor necrosis factor receptor associated factor 6, Treg – regulatory T cells, UC – ulcerative colitis, WBC – white blood count

miR	No. of source	Disease	Function/Interactions	Reference
**10a**	1	RA	Acts as a switch to control NFkB/YY1/miR-10a/NFkB regulatory circuit; downregulated in RA comparing to OA	[64]
	2	RA	Targets genes in TNF/IL-1 pathway (TAK1, IRAK4, BTRC); markedly reduced in RA FLS	[11]
**16**	1	JIA	Central regulator of TLR-mediated inflammatory response	[14]
	2	RA	Plasma levels inversely correlated with tender joint count and DAS28; upregulated in SF (compared to OA)	[15]
	3	JIA	Upregulated in JIA (especially polyarticular subtype), correlated with IL-6 and hip MRI scores	[17]
	4	RA	May become a predictor of a disease outcome in early RA (increased within the first 3 months and positively correlated with improvement in disease activity in subsequent 9 months)	[18]
	5	AS	Correlated with disease activity level	[20]
	6	RA	Altered in early RA compared to established RA or healthy controls	[24]

	7	RA	Correlated with DAS28, overexpressed in RA PBMCs (compared to OA)	[31]
	8	JIA	Targets IL-6, correlated with MMP3	[62]
	9	RA	Correlated with ESR, CRP, DAS28	[65]
	10	RA	Attenuation of macrophage-mediated pro-inflammatory response with subsequent improvement of insulin sensitivity	[66]
	11	RA	Upregulated in PBMCs, positively correlated with CRP and DAS28	[67]
	12	RA	May be associated with Th17/Treg imbalance through its effect on RORγt and foxp3	[68]
**17**	1	RA	Downregulated in serum and synovial tissues	[4]
	2	RA	Negative regulator of TNF signaling	[69]
**19a**		JIA	Positive regulator of JAK/STAT pathway, negative regulator of TNF and SOCS3 expression	[70]
**21**	1	JIA	Targets TNF expression	[70]
	2	RA	Targets programmed cell death 4, leading to IL-10 increase and NFkB decrease	[71]
	3	JIA	Promotes Th2 differentation	[72]
**22 (22-3p)**	1	RA	A part of a bioinformatic panel differentiating RA from controls	[5]
	2	RA	Associated with developing RA in individuals with positive ACPA	[73]
**23a**		RA	Correlated with response to ADA+MTX therapy in early RA	[55]
**23b**	1	RA	Positively correlated with ESR, CRP and DAS28; upregulated in ANA-positive RA	[51]
	2	RA	Promoter of Treg differentiation	[74]
**24 (24-3p)**	1	RA	A part of a bioinformatic panel differentiating RA from controls	[5]
	2	RA	Altered in early RA compared to established RA or healthy controls	[24]
	3	RA	Correlated with disease activity level	[75]
**26a**	1	JIA	Targets IL-6; upregulated in SoJIA, correlated with inflammatory severity	[50]
	2	JIA	Involved in bone erosion pathway through regulation of osteoclasts formation	[63]
**27a**		RA	Downregulated in RA FLS	[76]
**29a-3p**		AOSD	A part of bioinformatic panel predicting the probability of developing AOSD	[77]
**34a**		JIA	Upregulated in polyarticular JIA	[42]
**96-5p**		RA	A part of a bioinformatic panel differentiating RA from controls	[5]
**101-3p**	1	AOSD	A part of bioinformatic panel predicting the probability of developing AOSD; correlated with serum IL-6 and TNF	[77]
	2	RA	Reduced joint oedema in rats by downregulating PTGS2	[78]
**103a (103a-3p)**		RA	Associated with developing RA in individuals with positive ACPA	[79]
**124**		RA	Downregulated in RA and other inflammatory disorders (SLE, SS and UC)	[58]
**125a (125a-5p)**	1	JIA	Upregulated in SoJIA, correlated with inflammatory severity; promotes M2b macrophages polarization	[21]
	2	JIA	Correlated with the degree of systemic inflammation (ferritin, WBC, PLT) but not joint involvement	[23]
	3	RA	Altered in early RA compared to established RA or healthy controls	[24]
**125b**	1	RA	Involved in B cell differentiation and TNF expression; correlated with response to RTX	[61]
	2	JIA	Inhibitor of Th17 differentiation; downregulated in PBMCs and CD4+ T cells	[80]
**126**		JDM	Downregulated in untreated patients with the short duration of symptoms	[81]

**127-3p**		JIA	Upregulated in polyarticular JIA	[42]
**132 (132-3p)**	1	RA	High diagnosability in differentiation between RA and OA	[15]
	2	JIA	Downregulated in JIA when compared to JSA or healthy controls	[17]
	3	RA	Correlated with response to MTX	[37]
	4	RA	Inhibitor of IL-1, IL-6 and IFNβ expression	[82]
**133a**	1	RA	Upregulated in RA FLS	[59]
	2	JIA	Activator of NLRP3 inflammasome, upregulated at onset of SoJIA	[83]
**134-5p**		RA	A part of a bioinformatic panel differentiating RA from controls	[5]
**135b-5p**		RA	Correlated with disease activity level and inflammatory severity	[84]
**139-3p**		RA	Correlated with RF titer and with response to biologic DMARDs	[85]
**140-3p**		RA	A part of a bioinformatic panel differentiating RA from controls	[5]
**141-3p**		AOSD	High diagnosability in differentiation between AOSD from sepsis	[77]
**142-5p**	1	RA	Upregulated in RA FLS	[59]
	2	AOSD	A part of bioinformatic panel predicting the probability of developing AOSD	[77]
**143**		RA	Correlated with IL-6 and IL-8 levels	[86]
**145**	1	JIA	Upregulated in oligoarticular JIA when compared to polyarticular JIA and SoJIA	[50]
	2	RA	Involved in bone erosion pathway through regulation of osteoclasts formation; upregulated in RA PBMCs and synovium	[87]
**146a (146a-5p)**	1	RA	Upregulated in SF comparing to OA; plasma levels inversely correlated with tender joint count	[15]
	2	JIA	Upregulated in JIA comparing to JSA and healthy controls; correlated with JADAS27	[17]
	3	JIA	Upregulated in SoJIA monocytes, correlated with inflammatory severity (systemic features, ferritin, WBC, PLT); targets INHBA	[28]
	4	JIA	Promoter of M2 macrophages polarization	[29]
	5	JIA	Modulates NFkB pathway through downregulating IRAK1, IRAK2 and TRAF6	[30]
	6	RA	Correlated with improvement in disease activity level	[32]
	7	JIA	Upregulated in Th1 and downregulated in Th2	[33]
	8	RA	Decreased in early RA compared to established RA	[34]
	9	JIA	Association between rs2910164 SNP and susceptibility to ERA	[35]
	10	AS	Association between rs2910164 SNP and susceptibility to AS	[36]
	11	RA	Correlated with response to MTX	[37]
	12	RA	Upregulated in RA FLS	[59]
	13	JIA	A part of synovial fluid miRNome which differentiates JIA from *K. kingae* septic arthritis	[60]
	14	JIA	Correlated with MMP3	[62]
	15	RA	Inhibitor of TNF expression through targeting TRAF6 and IRAK1	[88, 89]
	16	RA	Promoter of Th17 differentiation through targeting IL-17; intensely expressed in synovium with hyperplasia	[90]
	17	RA	Downregulated in peripheral Tregs	[91]
	18	PsA	Overexpressed in PBMCs	[92]

**155 (155-5p)**	1	RA	Upregulated in RA FLS; inhibitor of MMP3 expression	[2]
	2	JIA/RA	Upregulated in PBMCs in JIA and RA when compared to SLE and FMF	[10]
	3	RA	Plasma levels inversely correlated with tender joint count	[15]
	4	JIA	Upregulated in polyarticular JIA (without statistical significance)	[16]
	5	JIA	Plasma levels decreased in oligoarticular and polyarticular JIA as well as in JSA	[17]
	6	RA	Correlated with response to MTX	[37]
	7	JIA	Promoter of M1 polarization through targeting SOCS1	[38]
	8	RA	Overexpressed in CD68+ macrophages;<br>decreased in early RA sera compared to established RA	[40]
	9	RA	Promoter of class-switched plasma producing IgG1 through targeting transcription factor Pu.1	[41]
	10	AS	Correlated with disease activity level	[43]
	11	RA	Correlated with ESR, CRP and DAS28; overexpressed in PBMCs	[44]
	12	JIA	A part of synovial fluid miRNome which differentiates JIA from *K. kingae* septic arthritis	[60]
	13	RA	Overexpressed in RA synovium when compared to OA	[93]
**181c**		JIA	Upregulated in active SoJIA; modulated CD163 expression in macrophages	[22]
**199a-5p**		PsA	Inhibitor of IL-6 and TNF expression; participates in autoregulatory loop along with NFkB;<br>involved in bone erosion pathway through suppressing MMP1	[94]
**204**	1	JIA	Downregulated in JIA patients with clinical remission on MTX monotherapy	[16]
	2	RA	Correlated with response to biologic DMARDs	[85]
**214**	1	RA	Downregulated in RA T cells	[85]
	2	RA/AS	Decreased in undifferentiated arthritis which was finally re-diagnosed as seronegative RA or peripheral AS	[95]
**223**	1	JIA	Upregulated in active SoJIA	[6]
	2	RA	Plasma levels inversely correlated with tender joint count	[15]
	3	JIA	Upregulated in JIA when compared to JSA or healthy controls	[17]
	4	RA	Serum levels correlated with CRP, DAS28 and change in disease activity after 3 and 12 months of follow-up	[18]
	5	RA	Overexpressed in naive CD4+ T cells and downregulated in Th17 cells	[24]
	6	JIA	Promoter of M2 macrophages polarization	[46]
	7	JIA	Inhibitor NLRP3 inflammasome	[47]
	8	RA	Correlated with RF titer	[48]
	9	CIA	Correlated with disease severity of murine CIA	[49]
	10	RA	Upregulated in RA FLS	[59]
	11	RA	Associated with disease relapse	[96]
**346**		RA	Upregulated in RA FLS	[59]
**361-5p**		RA	Upregulated in early RA	[7]
**379**		JIA	Upregulated in polyarticular JIA	[42]
**383**		RA	Associated with response to MTX, SSZ and GCS	[85]
**409-3p**		JIA	Upregulated in polyarticular JIA	[42]

**432-5p**	1	RA	Correlated with response to tofacitinib	[96]
	2	RA	Associated with determination of cardiovascular risk in RA	[97]
**448**		RA	Upregulated in RA and other inflammatory disorders (SLE, SS and UC)	[58]
**494**		JIA	Upregulated in polyarticular JIA (as well as in CF)	[42]
**551a**		JIA	Upregulated in polyarticular JIA (as well as in CF)	[42]
**551b**		RA	Upregulated in RA and other inflammatory disorders (SLE, SS and UC)	[58]
**627-5p**		RA	A part of a bioinformatic panel differentiating RA from controls	[5]
**760**		RA	Correlated with response to leflunomide and biologic DMARDs	[85]
**933**		JIA	Upregulated in polyarticular JIA	[42]
**936**		JIA	Downregulated in polyarticular JIA	[42]
**1285**		JIA	Upregulated in polyarticular JIA	[42]
**5571-3p**		RA	Correlated with disease activity level and inflammatory severity	[84]
**6764-5p**		JIA	A part of synovial fluid miRNome which differentiates JIA from *K. kingae* septic arthritis	[60]
**let-7a (let-7a-5p)**	1	RA	Correlated with DAS28	[32]
	2	RA	Stimulates formation of FLS	[98]
